# Chromatic transitions in the emergence of syntax networks

**DOI:** 10.1098/rsos.181286

**Published:** 2018-12-12

**Authors:** Bernat Corominas-Murtra, Martí Sànchez Fibla, Sergi Valverde, Ricard Solé

**Affiliations:** 1 Institute of Science and Technology Austria, Am Campus 1, 3400, Klosterneuburg, Austria; 2 Technology Department, Universitat Pompeu Fabra, Roc Boronat 138, 08018 Barcelona, Spain; 3 Complex Systems Lab, ICREA-Universitat Pompeu Fabra, Dr Aiguader 88, 08003 Barcelona, Spain; 4 Evolution of Technology Lab, Institut de Biologia Evolutiva (CSIC-UPF), Passeig Maritim de la Barceloneta, 37-49, 08003 Barcelona, Spain; 5 Santa Fe Institute, 1399 Hyde Park Road, Santa Fe, NM 87501, USA

**Keywords:** complex networks, graph colouring, modularity, syntax

## Abstract

The emergence of syntax during childhood is a remarkable example of how complex correlations unfold in nonlinear ways through development. In particular, rapid transitions seem to occur as children reach the age of two, which seems to separate a two-word, tree-like network of syntactic relations among words from the scale-free graphs associated with the adult, complex grammar. Here, we explore the evolution of syntax networks through language acquisition using the *chromatic number*, which captures the transition and provides a natural link to standard theories on syntactic structures. The data analysis is compared to a null model of network growth dynamics which is shown to display non-trivial and sensible differences. At a more general level, we observe that the chromatic classes define independent regions of the graph, and thus, can be interpreted as the footprints of incompatibility relations, somewhat as opposed to modularity considerations.

## 1. Introduction

The origins of human language have been a matter of intense debate. Language is a milestone in our evolution as a dominant species and is likely to pervade the emergence of cooperation and symbolic reasoning [1–4]. Maybe the most defining and defeating trait is its virtually infinite generative potential: words and sentences can be constructed in recursive ways to generate nested structures of arbitrary length [3,5]. Such structures are the product of a set of rules defining syntax, which are extracted by human brains through language acquisition during childhood after a small sample of the whole combinatorial universe of sentences has been learned. And yet, in spite of its complexity, syntax is accurately acquired by children, who master their mother tongue in a few years of learning. Indeed, around the age of two, linguistic structures produced by children display a qualitative shift on their complexity, indicating a deep change on the rules underlying them [6–8]. This sudden increase of grammar complexity is known as the *syntactic spurt*, and defines the edge between the *two words* stage, where only isolated words or combinations of two words occur, to a stage where the grammar rules governing this syntax are close to the one we can find in adult speech—although the cognitive maturation of kids makes the semantic content or the pronunciation different from the adult one. How can we explain or interpret such nonlinear pattern?

Statistical physicists have approached the problem of language evolution showing, for example, that non-trivial patterns are shared between language inventories—collections of words—and some genetic and ecological neutral models [9] (see [10] and references therein). However, most of these models do not make any assumption about the role played by actual interactions among words, or, more generally, linguistic units, which largely define the nature of linguistic structures. In this context, a promising approach to its structure and evolution involves considering language in terms of networks of interconnected units instead of unstructured collections of elements—e.g. words or syllables [11–15]. In this context, syntactic networks, in which nodes are words and links the projection of actual syntactic relations, have been shown to be an interesting abstraction to grasp general patterns of language production [7,8,16]. Specially valuable has been the quantitative data obtained from syntax networks obtained along the process of syntax acquisition, for they provided solid and quantitative evidence of sudden qualitative shifts in the cognitive machinery involved in the process, present also in other linguistic domains [7,8,16–19].

At the fundamental level, syntax can be understood as a set of symbols associated under a universe of potential combinations somewhat similar to chemistry. Atoms and words would then be linked through compatibility relations defining what can be combined and what is forbidden. The power of this picture is supported by the use of linguistic methods in the systematic characterization of chemical structures [20]. Chemical structure diagrams can thus be seen as some sort of language, with chemical species and bonds as key ingredients. In a more abstract fashion, we can say that general rules of combining elements within a given set of interacting pieces with well-defined functional meaning is at work in both language and chemistry. Following the chemical analogy, where abstract classes of ‘nodes’ can be defined, we will take advantage of graph colourability theory as a general framework to detect transitions based on qualitative changes of compatibilities. Specifically, we suggest that a new combinatorial approach grounded on graph colouring may enable a better understanding of the evolution of networks having internal relations of compatibility—e.g. some kind of syntactic rules. In this context, we propose the chromatic number—and complementary measures—of the graph [21–23] as an indicator of network complexity. The chromatic number is defined as the minimal number of *colours* needed to *paint* all nodes of the graph in a way that no adjacent nodes have the same colour [22]. In other words, classes of nodes would be defined precisely by the fact that there are no connections among them, *a measure conceptually opposite to graph modularity*. The *q*-colouring problem, i.e. to know whether a graph can be coloured with *q* different colours, is one of the most important *NP*-complete problems. From the statistical physics point of view, an analogous problem is defined within the context of the *Potts* model [24]. Transitions in the evolution of the chromatic number, which is the main objective of this work, have been widely studied in abstract models of random graphs [23,25–27].

The chromatic number may convey structural information among the classes of relations the graph is showing within the system it aims to abstract. This is exactly the point by which graph colouring is relevant for syntactic phenomena. We will work with a graph of aggregated syntactic relations using the paradigm of *dependence grammar* [28], which provides a natural framework to extract graphs from syntactic relations [7,8]. The aim of using the chromatic number comes from the intuition that syntactic relations do not glue elements *for free* but display consistent rules of compatibility/incompatibility among lexical elements. This may seem obvious at the level of the sentence analysis, but to extrapolate how these combinatorial rules among different classes of elements work at the global system level is a hard task, and even harder, if we want to do it quantitatively. For example, to grasp the relevance of chromatic number, one must perform parallel measurements on the network using indicators taking into account potential deviations—which will be the footprints of the non-trivial compatibility relations. The interplay between the evolution of the chromatic number and its deviations from a null model made of free associations will be the target of our paper. As we shall see, non-trivial transitions between different, increasing chromatic numbers, along with interesting deviations from a null model of syntax-free sentence generation are identified. This is, to the best of our knowledge, the first time that such transitions have been reported in a real system.

## 2. Graphs and colouring: basics

We will work over undirected graphs. An undirected graph G(V,E)—hereafter, G—is composed by the set of *V* = {*v*_1_, …, *v*_*n*_} *nodes* and a set *E* = {*e*_*j*_‖1 ≤ *j* ≤ *m*}⊆ *V* × *V* of edges. Each (unordered) pair *e*_*j*_ = {*v*_*i*_, *v*_*k*_} depicts a link between nodes *v*_*i*_ and *v*_*j*_. The number of links *k*(*v*_*i*_) attaching node *v*_*i*_ is the degree of the node and 〈*k*〉 is the *average degree* of the graph G. The *degree distribution* *P*(*k*) accounts for the probability to select a node at random having degree *k*. The identity card of a graph is the so-called *adjacency matrix*, a(G), which is defined as follows:

2.1 $$a_{ij}=\left\{ \begin{array}{rl} 1,\; & \text{iff}\;(\exists e_{k}\in E)\;:\;(e_{k}=\{v_{i},v_{j}\})\\ 0,\; & \text{otherwise}. \end{array}\right.$$

We observe that the adjacency matrix of undirected graphs is symmetrical, i.e. *a*_*ij*_ = *a*_*ji*_.

We can map the chromatic problem into the antiferromagnetic *q*-dimensional Potts model at *T* = 0 [24]. This model is a generalization of the classical Ising model for lattices: at every node of this lattice we place a particle having a spin which energetically constrains the state of its neighbours. Traditionally, spins can have only two states, namely | ↑ 〉 and | ↓ 〉. In the Potts model, compatibility relations take into account an arbitrary number *q* > 2 of different states. Let us consider a partition of nodes *V* containing *q* different classes, namely, *G*_*q*_(*V*) = {*g*_1_, …, *g*_*q*_} of *V*, i.e.

2.2 $$⋂G_{q}=\text{Ø}\;\text{and}\;⋃G_{q}=V,$$

The *state* σ_*i*_ of node *v*_*i*_ indicates the class of *G*_*q*_(*V*) to which the node belongs, i.e. σ_*i*_ ∈ *g*_*j*_. Let $F_{q}(V)$ be the ensemble of all partitions of *V* containing *q* different classes. Every element in $F_{q}(V)$ has the following energy penalty^1^:

2.3 $$H(G_{q})=J\sum_{i<j}a_{ij}\delta (\sigma_{i},\sigma_{j}),$$

where *J* = 1 is the *coupling constant* and *δ* is the Kronecker symbol

2.4 $$\delta (\sigma_{i},\sigma_{j})=\left\{ \begin{array}{rl} 1, & \text{iff}\;i=j\\ 0, & \text{otherwise}. \end{array}\right.$$

Intuitively, the higher the presence of pairs of connected nodes belonging to the same state, the higher will be the energy of the global state of the graph. Given a fixed *q*, the configurations displaying minimal energy may have an amount of non-solvable situations, leading to the unavoidable presence of connected nodes at the same state. This phenomenon is called *frustration*, and for these configurations, the ground state of the Hamiltonian defined in (2.3) displays positive energy. If there is no frustration, i.e. $\exists G_{q}\in F_{q}(V)$, we can find a partition that satisfies

2.5 $$H(G_{q})=0,$$

and we say that the graph is *q*-colourable, being the *q* different *colours* the *q* different classes or members of *G*_*q*_. When the graph is *q*-colourable, there is at least one partition $G_{q}\in F_{q}(V)$ such that, if *v*_*i*_, *v*_*j*_ ∈ *V* belong to the same *class* or *colour* of the partition, namely *g*_*l*_ ∈ *G*_*q*_. We deduce that

2.6 $$(v_{i},v_{j}\in g_{l})\;\Rightarrow \;a_{ij}=0.$$

Relation (2.6) maps colour classes onto disjoint sets of graph elements (adjacent nodes have a different colour). Now, the colouring problem consists in finding the minimal number of classes (or colours) required to properly *paint* the graph. This is the so-called *chromatic number* of the graph G

2.7 $$\chi (G)=\min\{q\;:\;(\exists G_{q}\in F_{q}(V))\;:\;H(G_{q})=0\}.$$

Now suppose network partition(s) $G_{q}^{*}\in F_{q}(V)$ having minimal energy, see equation (2.3), given a number of colours *q*

2.8 $$G_{q}^{*}=\min_{G_{q}\in F_{q}(V)}\{H(G_{q})\}.$$

In general, the process of search for the chromatic number yields a decreasing sequence of energies ending at $H(G_{\chi (G)}^{*})=0$

2.9 $$H(G_{1}^{*})\leq \cdots \leq H(G_{\chi (G)}^{*})=0,$$

In order to assess the statistical significance of chromatic numbers, we define the *relative energy* of any *q*-colouring as follows:

2.10 $$f_{q}(G_{q}^{*})=\frac{H(G_{q}^{*})}{\left|E\right|},$$

where |*E*| is the number of edges in the graph *G*. This quantity $0\leq f_{q}(G_{q}^{*})\leq 1$ corresponds to the minimal (relative) number of frustrated links or *violations* (i.e. when adjacent nodes have the same colour).

Despite the high complexity of this problem—computing the chromatic number in an arbitrary graph is a *NP*-hard problem—several bounds can be defined. A lower bound can be defined from the so-called *clique number*. A *clique* is a subgraph in which every node is connected to all other nodes in the subgraph. The *clique number* ω(G) is the size of the largest clique in the graph, which is a natural lower bound for χ(G) [22]

2.11 ω(G)≤χ(G).

Alternatively, an upper bound on χ(G) can be defined by looking at the *K*-core structure of G. The K(G) core is the largest subgraph whose nodes display degree higher or equal to *K*. Now, let $K^{*}(G)$ be the *K*-core with largest connectivity that can be found in G

2.12 $$K^{*}=\max\{K\;:\;K(G)\neq \text{Ø}\}.$$

Then, it can be shown that $K^{*}$ sets an upper bound to the chromatic number [22]

2.13 $$\chi (G)\leq K^{*}+1.$$

Finally, let us mention that, for some families of random graphs the chromatic number has an asymptotic behaviour depending on the average connectivity [23], χ(G)∼⟨k⟩/log⁡⟨k⟩. However, the above relationship does not hold for scale-free networks with exponent 2 < γ < 3. These heterogenous networks cannot have a stable value of the chromatic number because their clique number (2.11) diverges with the graph size, even at constant 〈*k*〉 [29].

## 3. The evolution of *χ* along syntax acquisition

Here, we study the evolution of the chromatic number through language development as captured by syntax graphs. We compare the chromatic number with the lower and upper bounds provided by the clique number and the maximal *K*-core, respectively. We assess the relevance of computed chromatic numbers with the corresponding minimal energy. The combination of these two measurements enable us to interpret the nature of the chromatic number. Specifically, we can check whether changes in this number reflect a global pattern or instead some anomalous behaviour of a small, localized subgraph. Finally, we provide further validation of our analysis by comparing chromatic numbers in empirical and synthetic networks obtained through a random sentence generator.

### 3.1. Building the networks of early syntax

Through the process, networks built upon the aggregation of syntactic structures from child’s productions grow and change in a smooth fashion until a rapid transition occurs [7,8,30,31] (see also [13]). We reconstruct syntactic networks by projecting the raw constituent structure, i.e. phrase structure of children’s utterances, into linear relations among lexical items, in what is known as *dependency grammar* analysis [28,32]. Then, we aggregate all these productions in a single graph where nodes are lexical items and links represent syntactic relations between them [7,30,31]. We emphasize that these networks have been built *by hand*, in the sense that no automatic procedure has been at work. The reason stems from the fact that early child language is far from normative, but, still, structures can be identified. Therefore, each link is discussed after checking its suitability according to specific linguistic criteria developed for this analysis (see [7,30,31] and references therein). These networks provide a unique window into the patterns of change occurring in the language acquisition process.

The two cases studied here are obtained from the CHILDES database [33,34] which includes conversations between children and parents. Specifically, we choose Peter and Carl’s corpora, whose structure has been accurately extracted and curated. For both Peter and Carl’s corpora, we choose 11 different recorded conversations distributed in approximately uniform time intervals ranging from the age of approximately 20 months to the age of approximately 28 months. The chosen interval corresponds to the period in which the syntactic spurt takes place. From every recorded conversation, we extract the syntactic network of child’s utterances obtaining a sequence of 11 syntactic graphs corresponding to the sequence of Peter's conversations $G_{P1},\ldots ,\;G_{P11}$ and Carl’s conversations $G_{C1},\ldots ,\;G_{C11}$.

### 3.2. Chromatic transition from bipartite to multicoloured networks

From our graph collection (see §3.1), we obtain two sequences of chromatic numbers *s*_*P*_(*χ*) and *s*_*C*_(*χ*) corresponding to the evolution of the chromatic number in Peter and Carl datasets, respectively:

$$\begin{array}{rl} s_{P}(\chi ) & =\chi (G_{P1}),\ldots ,\;\chi (G_{P11})\\ s_{C}(\chi ) & =\chi (G_{C1}),\ldots ,\;\chi (G_{C11}). \end{array}$$

**Figure 1.**
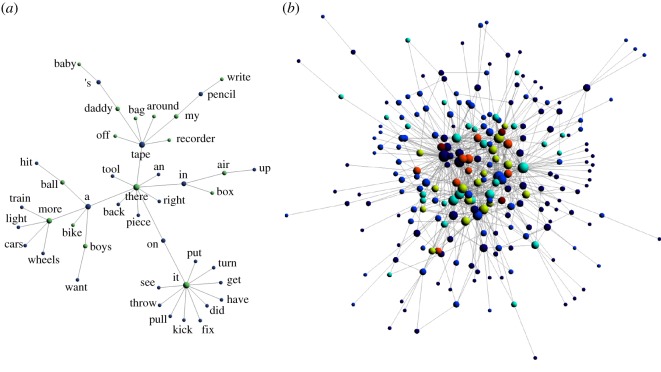
Optimal colourings of syntactic networks before and after the syntactic spurt. (*a*) A syntactic network before the transition (3rd corpus) is largely bipartite (this network accepts a 2-colouring). (*b*) Post-transition network (7th corpus) is remarkably more complex, which corresponds to high chromatic number $\chi (G_{7})=6$. All networks coming from Peter dataset. Time spent between these two corpora is about two and a half months—see text.

**Figure 2.**
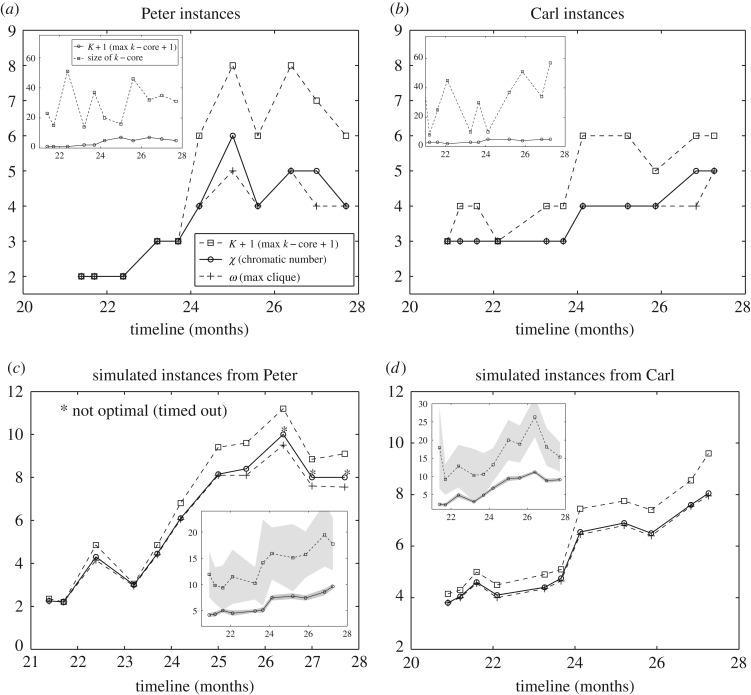
Evolution of the chromatic number *χ* (solid line), maximum clique *ω* (dashed line with crosses) and the maximum core $K^{*}+1$ (dashed line with squares) in Peter’s corpus (*a*) and Carl’s corpus (*b*). Evolution of the same measures over an ensemble of *n* = 20 networks obtained after running the null model fed with Peter’s corpus data (*c*) and Carl’s corpus (*d*). Insets: comparison between the time evolution of the deepest *K*-core, $K^{*}$-core, size (dashed line)—i.e. number of nodes within this subgraph—and the $K^{*}$ corresponding to the deepest *K*-core (solid line) for each set of networks. Shaded grey areas correspond to standard deviation in the case of the simulated instances.

The above sequences display similar patterns with some interesting differences (figures 2 and 4). For example, the middle stages of both datasets show an increase in the chromatic number. At the stage when the syntactic spurt takes place, Peter’s dataset *s*_*P*_ displays a sharp transition from a nearly constant, low chromatic number (*χ* = 2 up to just before month 23) to a high chromatic number (up to *χ* = 6, month 25) which is fully consistent with the emergence of complex syntax. The first three networks in *s*_*P*_ accept 2-colourings, i.e., they are bipartite, see figure 1.

The grammar at this stage mainly generates pairs of complementary words, like:

$$\begin{array}{rl} & \langle \text{verb, noun}\rangle \;\text{or}\\ & \langle \text{adjective, noun}\rangle . \end{array}$$

Typical productions of this stage are, for example, ‘car red’ or ‘*horsie* run’. This pre-transition pattern, also called 2-word stage, corresponds to a highly restrictive grammar, e.g. syntactic structures like 〈verb, verb〉 do not exist. Instead, relations between lexical items are strongly constrained by their semantic content. On the other hand, Carl’s sequence *s*_*C*_ shows *χ* ≥ 3 from the very beginning—i.e. these networks are not bipartite. A detailed inspection of Carl’s productions at this stage shows the presence of functional particles from the very beginning. Functional particles are those lexical items whose role is essentially grammatical, and whose appearance must be accompanied by another, strongly semantic word, like a noun or a verb. We consider as *functional particles* the set of lexical items composed by articles—like *a* or *the*, prepositions—like *at* or *with*, auxiliary verbs—like *do* or *will*, when they accompany another verb. The presence of functional particles from the very beginning in Carl’s corpus suggests that, in general, high chromatic numbers relate to high grammar flexibility, this flexibility being provided by the *hinge* role that these particles have in the global functioning of grammar.

Still, the behaviour of the chromatic number of a graph χ(G) can be quite sensitive to the anomalous behaviour of small subgraphs. For example, the transition of $\chi (G_{2})=2$ to $\chi (G_{3})=3$, when Peter is about 23 months old, is due to a single triangle in a (largely) bipartite network (figure 2*a*,*c*). A combination of measurements enables us to assess whether the chromatic number represents the behaviour of a small number of nodes or is the natural outcome of global network features. Our choice is to compare χ(G) with the lower bound given by the clique number (2.11) and the upper bound provided by the maximal *K*-core connectivity (2.12). Therefore, each sequence *s*_*P*_(*χ*), *s*_*C*_(*χ*) will be accompanied by two sequences, namely *Ω*, *κ*

$$\begin{array}{rl} \Omega_{P,C} & =\omega (G_{P1,C1}),\ldots ,\;\omega (G_{P11,C11})\\ \kappa_{P,C} & =K^{*}(G_{P1,C1}),\ldots ,\;K^{*}(G_{P11,C11}). \end{array}$$

For example, figure 2*a*,*b* shows a clear increasing trend both for maximum clique and maximum *K*-core. This, combined with the sequence of energy values given in table 1, indicates that the final chromatic number can no longer be associated with any trivial clique or a tiny fraction of the maximum *K*-core. Both Peter and Carl sequences show that the chromatic number is often close to the clique number (figure 2*a*,*b*). Maximum $K^{*}$-core size is generally more than twice the size it would have in the case that it would form a clique—see figure 2 (inset). We therefore conclude that an important part of the whole network structure has enough connectivity to enable the emergence of a non-trivial *K*-core structure. The whole picture points towards the existence of a broad connectivity pattern responsible for the emergence of increasing chromatic numbers. Nevertheless, even acknowledging the role and suitability of the indicators of validity for the chromatic number used here—maximum *K*-core, maximum clique number and sequences of *relative energy* of successive colorations of the graph—one cannot completely rule out the existence of a pathological, largely statistically deviated small set of nodes responsible for the behaviour of the chromatic number. We observe that a conclusive response would involve the analysis of the combinatorics among all subsets of the network, which defines a computationally unaffordable problem. We warn the reader that this problem is not restricted to the chromatic number, but it may be present in almost any network measure.

**Table 1.** Relative energy values of *q*-colourings in the Peter (top) and Carl (bottom) datasets. Relative energies reveal the fraction of frustrated links in the optimal colouring using *q* different colours.

	$G_{P1}$	$G_{P2}$	$G_{P3}$	$G_{P4}$	$G_{P5}$	$G_{P6}$	$G_{P7}$	$G_{P8}$	$G_{P9}$	$G_{P10}$	$G_{P11}$
$f_{1}(G_{1}^{*})$	1	1	1	1	1	1	1	1	1	1	1
$f_{2}(G_{2}^{*})$	0	0	0	1/49	5/105	66/434	131/644	87/589	157/903	104/659	95/717
$f_{3}(G_{3}^{*})$	0	0	0	0	0	8/434	31/644	15/589	40/903	20/659	10/717
$f_{4}(G_{4}^{*})$	0	0	0	0	0	0	8/644	0	8/903	2/659	0
$f_{5}(G_{5}^{*})$	0	0	0	0	0	0	1/644	0	0	0	0

	$G_{C1}$	$G_{C2}$	$G_{C3}$	$G_{C4}$	$G_{C5}$	$G_{C6}$	$G_{C7}$	$G_{C8}$	$G_{C9}$	$G_{C10}$	$G_{C11}$
$f_{1}(G_{1}^{*})$	1	1	1	1	1	1	1	1	1	1	1
$f_{2}(G_{2}^{*})$	6/140	5/119	11/156	6/128	10/152	14/199	61/361	65/442	71/439	93/592	131/687
$f_{3}(G_{3}^{*})$	0	0	0	0	0	0	9/361	11/442	8/439	16/592	29/687
$f_{4}(G_{4}^{*})$	0	0	0	0	0	0	0	0	0	1/592	4/687

### 3.3. Real syntax versus null model

Here, we compare the evolution of the chromatic number in real and simulated networks. A data-driven, syntax-free model that generates random child’s utterances having the same statistics of word production as Peter and Carl datasets is used as a null model [7]. Underlying the null model outlined below, there is the aim to *reproduce a syntax-free speech flow*, with the same statistical indicators as the real data. That means that we prioritized the simulation of a speaker whose statistics over words usage and sentence length mimic the ones given by the data. Different realizations of the model may lead, for example, to slightly different number of used words—for it is a stochastic phenomena with fluctuations at the sizes we are working in. This is due to the fact that our aim has been, not to randomize the network itself—which would have been the standard approach—but *the process that creates the network*. Since the network is a surrogate of an underlying phenomenon, it is more realistic to create a random version of such underlying phenomenon and, then, build the network, than randomize the network itself. This model definition enables us to assess if the high combinatorics displayed by post-transition networks emerge directly from an increasingly rich vocabulary. We build our model by extracting the following statistical parameters from the 11 recorded conversations in Peter and Carl corpora:

(1) The number of sentences |*S*_*P*_(*i*)|, *S*_*C*_(*i*) in the Peter and Carl datasets.
(2) The probability distribution of *structure lengths* or the probability *P*(*s*) that any syntactic structure has *s* words. We obtain two different distributions, one for each dataset.
(3) We assume that the probability of the *i*th most frequent word is a scaling law
  3.1 $$p(i)=\frac{1}{Z}i^{-\beta },$$
  with 1 ≤ *i* ≤ *N*_*w*_(*T*), *β* ≈ 1—i.e. Zipf’s Law—and *Z* is the normalization constant
  3.2 $$Z=\sum_{i=1}^{N_{w}(T)}{\left(\frac{1}{i}\right)}^{\beta }.$$
  Note that *Z* depends on lexicon size, *N*_*w*_(*T*), which grows slowly at this stage.

**Figure 3.**
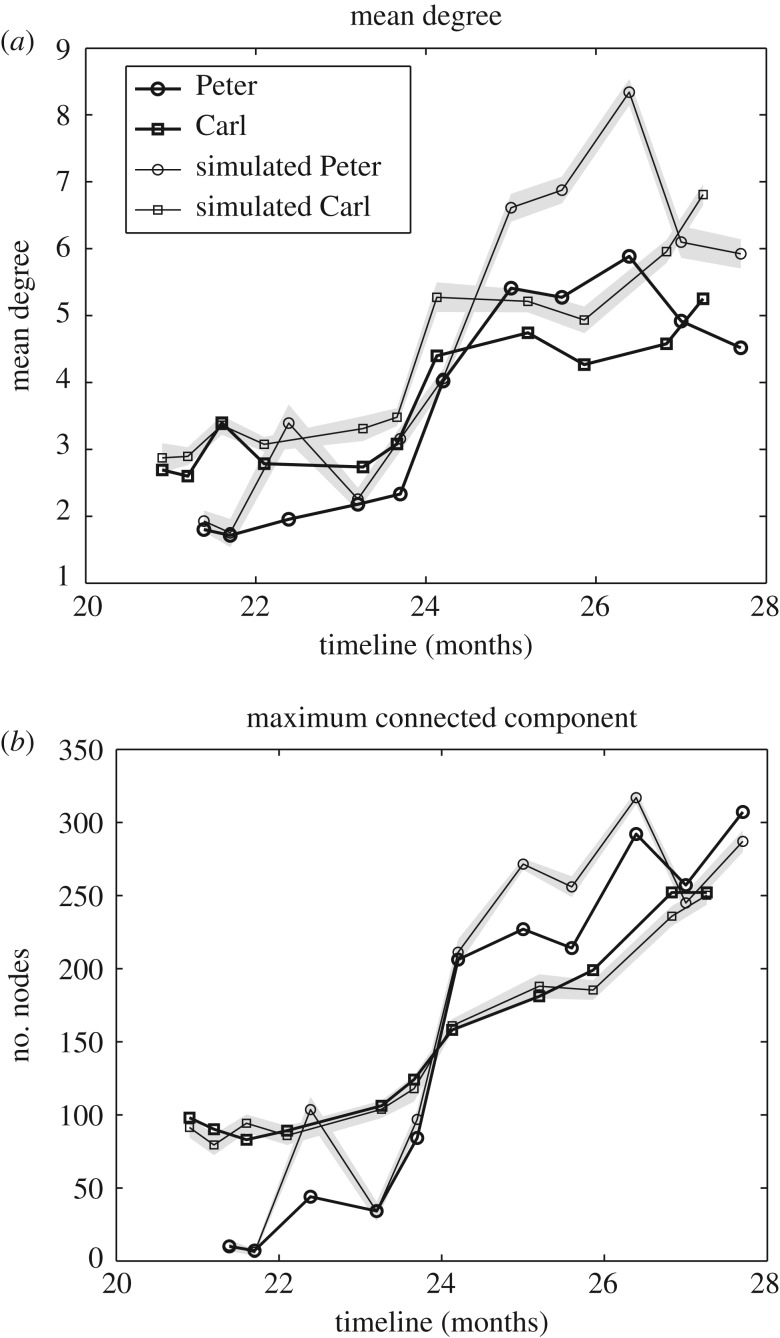
Evolution of (*a*) the mean degree and (*b*) size of the largest connected component in the real (strong solid lines) and simulated (weak solid lines) syntax networks. Shaded grey areas correspond to standard deviation in the case of the simulated instances.

We run the above model in the two datasets by generating |*S*_*P*,*C*_(*i*)| random sentences, each experiment is repeated 20 times. From the collection of randomly generated syntactic structures we construct a comparable sequence of syntax networks following the same method as in the real datasets (see §3.1). Figure 3 shows that our model generates random syntax networks with size and connectivity comparable to the ones measured in real networks. These statistical indicators display a huge increase during the studied period, this increase being sharper around the age of two, i.e. during the syntactic spurt [7]. As discussed in §2, both the mean connectivity and network size play an important role when determining the values of *ω*, *χ* and $K^{*}$.

Now, we compute the sequence of averaged chromatic numbers, ${\tilde{s}}_{P}(\chi ),{\tilde{s}}_{C}(\chi )$, for the simulated Peter and Carl syntax networks. Similarly, we generate the sequences of average clique number ${\tilde{\Omega }}_{P,C}$ and the average maximum *K*-core ${\tilde{\kappa }}_{P,C}$. The most salient property we find when comparing real networks obtained from both Peter and Carl’s corpora with their randomized counterparts is a huge increase of *χ*, *ω* and $K^{*}$ in the simulated networks. That is, the ensemble of random strings displays higher complexity parameters than the real corpora. For example, at the end of the studied period, the three complexity estimators are close to 10 in Peter simulations and close to 9 in Carl simulations (figure 2*c*,*d*).

**Figure 4.**
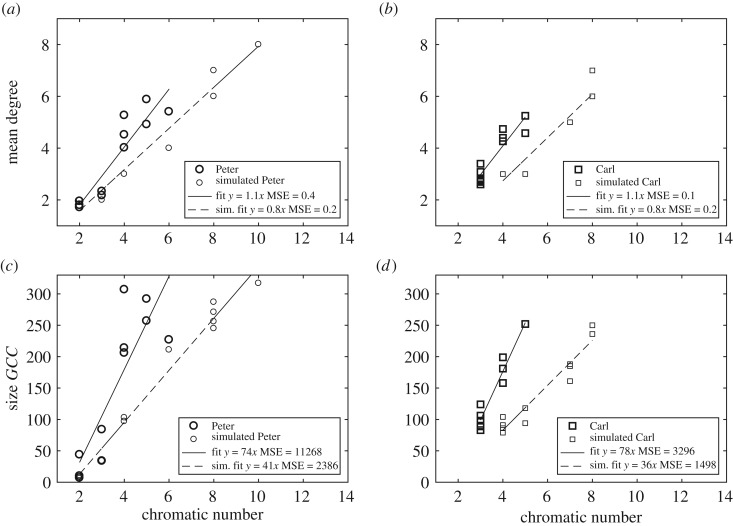
Relationship between the chromatic number, mean degree and giant connected component (*GCC*), comparing real data from the null model. (*a*) Scatter plot between the chromatic number and the average connectivity of the networks for Peter’s corpus and its associated null model. (*b*) Scatter plot between the chromatic number and the average connectivity of the networks for Carl’s corpus and its associated null model. (*c*) Scatter plot between the chromatic number and the size of the *GCC* for Peter’s corpus and its associated null model. (*d*) Scatter plot between the chromatic number and the size of the *GCC* for Carl’s corpus and its associated null model. Clearly, all indicators show that the chromatic number observed in real graphs is below that expected by a non-syntactic speaker.

A very interesting feature is found at the first stages of the simulated Peter sequence: the random networks are no longer bipartite—see §3.2. In particular, the third random corpus has an average chromatic number of 4, which is significantly higher than the observed chromatic number. In this case, the two-stage grammar imposes severe constraints on what is actually plausible in any pre-transition syntactic structure. This trend is also observed at later stages of language acquisition. In general, simulated networks have higher chromatic numbers than empirical networks, although both two types of networks have similar connectivities—by definition. In some cases, the average chromatic number of the graphs belonging to the random ensemble is twice the real one (figure 2). To better understand the nature of these deviations, we have compared the behaviour of chromatic numbers against mean connectivity and the size of the largest connected component. Figure 4 shows a well-defined, non-trivial deviation between real networks and random networks. In particular, when comparing the relation between the chromatic number and the average degree of Peter’s corpus (figure 4*a*) and Carl’s corpus (figure 4*b*) with the simulated ones, we observe a clear trend of the real networks towards smaller chromatic numbers. The comparison of the size of the giant connected component, *GCC*, clearly depending on the size in the case of scale-free networks [29], shows the same trend both in Peter’s (figure 4*c*) and Carl’s (figure 4*d*) corpora. In general, the expected chromatic number is larger than the one observed in the real networks. These plots suggest that the chromatic number is capturing essential combinatorial properties of the underlying system, which cannot be reproduced with a simple, syntax-free random generation model. We support this argument by providing a linear regression fit to the data. In the case of the relation of the *GCC* and the chromatic number, the mean squared error (MSE) is substantially higher in the case of the real instances, Peter and Carl (figure 4).

## 4. Discussion

Syntax is a characteristic, complex and defining feature of language organization. It pervades its capacity for unbounded generative power of the linguistic system [5], allows sentences to be organized in highly structured ways and is acquired in almost full power by children after being exposed to a limited repertoire of examples. Syntax is also one aspect of the whole: semantic and phonological aspects need to be taken into account, and they are all embedded in (and run by) a cognitive, brain-embodied framework [35]. Because of the dominant role played by how words actually interact with each other, computational and theoretical approaches dealing with word inventories or other statistical trends ignoring interactions are likely to be limited. As an example of the high degree of intricacy involved in linguistic acquisition, we mention two recent works: First, recent studies on acquisition in French toddlers provided strong evidences for non-trivial interactions between phonological, semantic and syntactic *modules*, showing the presence of inhibition/activation patterns in the acquisition dynamics involving cross-dependencies among those modules [36]. The second example comes from the framework of *multiplex networks*—i.e. networks involving different layers of interaction (see [37,38] and references therein). Specifically, it has been shown that, taking into account different layers of interactions, a critical phase can be identified from a simple, restricted grammar towards a flexible, more complex grammar [18,19], consistent with the results provided in our paper.

Following previous work that takes advantage of complex networks approaches to language organization [13] we have made a step further in studying the structure of syntax graphs using graph colouring. The motivation of this approximation is twofold. On the one hand, graph colourability allows to properly detect correlations that are not captured by topological approaches. On the other hand, it seems a natural way to substantiate previous claims connecting syntax with compatibility relations common with other types of systems, such as chemical structures. In this context, standard network measurements like average degree, clustering or degree distribution are much more limited. Since graph colouring naturally defines compatibility through the presence or absence of a common label to every pair of nodes, it seems the right framework to study the process of network growth in child language. The behaviour of the chromatic number accurately marks the syntactic spurt in language acquisition, i.e. it is a footprint of the generative power of the underlying grammar.

There are limitations associated with the network definition. Syntactic relations are structure-dependent, not sequence dependent. Because the network is an aggregation of text sequences, it cannot fully grasp the hierarchical nature associated with syntactic constructs. Still, the chromatic number is a global measurement that can detect grammar constraints by analysing the pattern of network interaction at different scales. That is, the network representation is an indicator of global linguistic proficiency and includes some combinatorial signal which can be properly detected with the chromatic number. Besides the suitability of the measure, it is in force to highlight that more longitudinal studies are needed. In this case, we studied two single individuals whose data is of excellent quality. Moreover, we assembled the syntactic networks by discussing the linguistic validity of each syntactic relation in detail. We therefore have chosen this high level of accuracy in our analysis, in spite of performing a massive one with less delicate assembling methodology. Further studies should perform much more longitudinal explorations, involving eventually other languages or bilingual/multilingual children, with the same degree of detail in the analysis, when possible.

There are other, broader implications of our work. The chromatic number can be viewed as a reciprocal measure of standard community detection. Here, the chromatic number defines a partition of the network in classes of unlinked nodes. This definition is particularly relevant in networks where some kind of compatibility relation is at work in the wiring process. In this case, the standard community structure can be misleading, because elements of the same class cannot be connected. The case for syntactic graphs is paradigmatic but the partition induced by the chromatic number could shed light into the behaviour of many other systems. Additionally, we have proposed to assess the statistical significance of these partitions with the sequence of minimal violations—see equation (2.10). Future work should explore how the chromatic number—and related measures—can be exploited to detect *forbidden* links in the network. Deviations of the chromatic number, as the ones observed in this paper, suggest the presence of combinatorial constraints that must be taken into account, for example, when defining proper null-models.

## Supplementary Material

Reviewer comments

## Footnotes

1 In our approach, the energy units of this Hamiltionian are arbitrary.

## Data accessibility

Data have been extracted from the CHILDES database. Transcripts from Peter’s corpus are found in: https://childes.talkbank.org/browser/index.php?url=Eng-NA/Bloom70/Peter/. Transcripts for Carl’s corpus are found in: https://childes.talkbank.org/browser/index.php?url=Eng-UK/Manchester/carl/.
