# Supplementary material for: Chromatic transitions in the emergence of syntax networks
Source: R Soc Open Sci. 2018 Dec 12;5(12):181286. doi: 10.1098/rsos.181286 (PMC6304139; doi:10.1098/rsos.181286)
Supplement: Reviewer comments [file rsos181286_review_history.pdf]

THE ROYAL SOCIETY
PUBLISHING

ROYAL SOCIETY
OPEN SCIENCE

Chromatic transitions in the emergence of syntax networks

Bernat Corominas-Murtra, Martí Sànchez Fibla, Sergi Valverde and Ricard Solé

Article citation details
R. Soc. open sci. **5**: 181286.
http://dx.doi.org/10.1098/rsos.181286

Review timeline
Original submission: 3 August 2018
Revised submission: 22 October 2018
Final acceptance: 29 October 2018

Note: Reports are unedited and appear as submitted by the referee. The review history appears in chronological order.

Review History

RSOS-181286.R0 (Original submission)

Review form: Reviewer 1 (Massimo Stella)

Is the manuscript scientifically sound in its present form?
Yes

Are the interpretations and conclusions justified by the results?
Yes

Is the language acceptable?
Yes

Is it clear how to access all supporting data?
Yes

Do you have any ethical concerns with this paper?
No

Have you any concerns about statistical analyses in this paper?
No

Reports © 2018 The Reviewers; Decision Letters © 2018 The Reviewers and Editors; Responses © 2018 The Reviewers, Editors and Authors. Published by the Royal Society under the terms of the Creative Commons Attribution License http://creativecommons.org/licenses/by/4.0/, which permits unrestricted use, provided the original author and source are credited

Recommendation?
Accept with minor revision (please list in comments)

Comments to the Author(s)
The authors apply the chromatic number to the investigation of the growth and development of the mental lexicon of English toddlers. The chromatic number is relative to the well studied problem of graph colouring, which has been linked to other instances of problems such as SAT problems and which are massively investigated in Statistical Physics and Computer Science.

The application of the chromatic number to word learning is therefore an interesting, elegant and clever point of novelty for detecting meso-scale changes in the structure of the mental lexicon of toddlers. The authors took a great deal of effort in explaining the details behind the chromatic number also to an audience outside of the physics realm, which is the right direction for such an interdisciplinary investigation in Complexity Science.

Although the paper is nicely structured, there are some points that should be addressed by the authors before publication. In particular, my main concern is about individual variability making it quite difficult to make general statements about sudden transitions in network structure during early word learning. This crucial point should be better discussed by the authors. I believe they should underline that the syntactic spurt they detect in two children has been intensively studied by previous studies and that the current technique is capable of highlighting this pattern in those specific children. I would like for the authors to discuss some more of the representativeness of the data analysed in the current manuscript, especially considered that previous network approaches have analysed datasets of longitudinal data accounting for individual variability.

Also, in order to improve the readability of the manuscript by also language scientists from the cognitive sciences, I suggested a few references about past approaches to modelling word learning as structural transition in networks of lexical items. I would recommend for the authors to integrate these references in the manuscript in order to improve the interpretation and stress by comparison novelty of their interesting results.

The references I pointed out all agree on indicating that network modelling of early word learning is quickly becoming a "hot" topic in the community of complex systems, hence the scope and timing of the manuscript would be ideal for an interdisciplinary journal venue like Royal Society Open Science.

I hope my comments provide useful feedback from the perspective of the cognitive sciences. I would be more than happy to review again this interesting manuscript.

Massimo Stella, PhD
Fondazione Bruno Kessler, Italy
Institute for Complex Systems Simulation, University of Southampton, UK

1) Page 1, Column 1, Line 51 – The authors talk about language evolution and then structure a brief literature review of models about it. However the main scope of the paper is language acquisition. Language evolution is a different process compared to language acquisition, happening at different time scales and at different levels of a given population of language speakers. I would recommend for the authors to explicitly underline that language learning is a different process compared to language acquisition and re-focus a bit the literature review over word learning rather than on language evolution.

2) I would suggest briefly mentioning two works.

One is by cognitive scientists and uses co-occurrence networks, which partially overlap with syntactic networks, for investigating word learning in typical and late talkers:
Beckage, N., Smith, L., & Hills, T. (2011). Small worlds and semantic network growth in typical and late talkers. PloS one,6(5), e19348.
The main result of the paper is that late talkers display different co-occurrence network features compared to normative learners. Hence, syntactic networks can capture real-world patterns of language learning.

Another suggestion fitting the scope of the paper would be an approach by statistical physicists and cognitive scientists in tracking network structural changes in a multiplex network of semantic, taxonomic and phonological word-features:
Stella, M., Beckage, N. M., Brede, M., & De Domenico, M. (2018). Multiplex model of mental lexicon reveals explosive learning in humans. Scientific reports, 8(1), 2259.
The main result of the paper is that the mental lexicon of normative talkers displays an explosive phase transition around age 7 yrs, a well documented age of increased cognitive and linguistic development. This is another work showing that linguistic transitions can indeed be captured by complex networks.
Enriching the introduction with a brief mention of other relevant network approaches would increase the novelty of the approach provided by the authors of this manuscript, by contrast/comparison.

3) Page 4, Column 2, Line 37 – "First tree networks…" -> "The first tree networks…"

4) Page 4, Column 2, Last Paragraph – Please explain what "functional particles" are for the non-experts by providing some examples. Also, what do the authors mean by high "grammar flexibility"? This part of the results is particularly relevant for the paper and should be described in more detail, with some examples.

5) Page 5, Column 2, Line 49 – The measure of relevance \chi was called relative energy previously. Repeating its name here would improve the clarity of this sentence.

6) Page 5, Column 2, Last Paragraph – What do the authors mean by "clear trend towards increasing maximum clique and maximum K-core with increased relevance"? How is relevance defined? This is an important statement for justifying how the chromatic number can be a valid proxy of "global" network features. Please reword this passage for increased clarity. The rewording has to be careful with using "global", since even showing the presence of larger K-cores does not really imply a global structural pattern.

I imagine a simple counter-example in which a fictional network densifies its core by only adding more links, so that its maximum K-core becomes larger and K increases. However, at the same time, the network could leave its periphery completely untouched. In that case, the sign of an increasing K would not be a global estimator of structural changes in the network but rather a sign of increasing connectivity at the meso-scale level of the network core. If this was the case also in the growing syntactic network, would it be better to say that the chromatic number is an estimator of meso-scale or non-local, rather than global, network organization? The authors leave this open to speculation later on line 7 of Page 6, where they say that rather than the whole network it might be just a part of it leading to the emergence of a non-trivial K-core structure.

7) Page 6, Column 1, Line 1 – What do the authors mean by saying that the relevance of the chromatic number as a global complexity estimator is much more "feasible"? Also the term "global complexity" can be ambiguous in this context, as it might refer to specific complexity measures from the cognitive sciences rather than to addressing the structural organization of a complex network. Please reword this passage for increased clarity.

8) Page 7, Figure 4. The authors claim in the text that Figure 4 shows a well-defined non-trivial deviation between real networks and random networks. However, provided the small number of samples, it is really difficult to see this in Figure 4, between top and bottom panels. I would suggest presenting at least correlation measures or other quantitative estimates for the correlations. Also, for visual inspection it would help to produce all the panels on the same ranges and improve the size of the points. Currently, it feels difficult for the reader to reconcile the text with the figure. This point is particularly important for the very interesting results reported in the text.

9) Page 7, Column 2, Line 41 – The authors talk about combining aspects of syntax, phonology and semantics. It would be of relevance to briefly discuss again two approaches, one from the cognitive sciences and one from physicists/computer scientists.

From the cognitive sciences, it could be of relevance the approach by Dautriche and colleagues. They modelled French word acquisition in toddlers of 18 months. Dautriche and colleagues reported evidence that contrary to previous conjectures, early word learning is not dominated by simple phonological similarities but rather by a complex multidimensional combination of phonological, syntactical and semantic similarities among words. Dautriche and colleagues reported that for nouns, these multi-dimensional similarities inhibited the acquisition of new nouns while network similarities facilitated acquisition of new verbs. These facilitatory/inhibitory effects might be causing the increased structural complexity captured by the authors through the chromatic number. The reference is:
Dautriche, I., Swingley, D., & Christophe, A. (2015). Learning novel phonological neighbors: Syntactic category matters.Cognition, 143, 77-86.

From physicists, it could be of relevance the approach by Stella and colleagues, who used networks of co-occurrences, free associations, feature sharing and phonological similarities for predicting early word learning on the same dataset used by the authors. Although Stella and colleagues did not use syntactic links but rather co-occurrences as a proxy for syntactic relationships, they also showed that: (i) month 23, close to the spurt investigated by the authors, is an important "critical" phase where children start using mainly free association for word learning; (ii) syntactic relationships are important throughout early development for predicting word learning, but only when the global structure of the mental lexicon is considered, in agreement with the message behind the chromatic number about the global structure of the mental lexicon being relevant to word acquisition. The reference is:
Stella, M., Beckage, N. M., & Brede, M. (2017). Multiplex lexical networks reveal patterns in early word acquisition in children.Scientific Reports, 7, 46730.

10) Page 8, Column 1, Line 15 – Rather than "linguistic performance" it would be more appropriate to say "linguistic proficiency".

11) Page 8, Column 1, Line 19 – The authors indicate the chromatic number as being evidently better in underlining the presence of the syntactic spurt. However, it should be underlined that the comparison has been performed only on two children. Such a small size constitutes a problem in making general statements and should be carefully addressed by the authors. Longitudinal studies over the same CHILDES dataset used by the authors have been testing many more children. For instance, Beckage et al. (2011) investigated word learning in 66 children. Why was the current analysis limited to only 2 children? Where there data limitations? The main issue here is that individual variability plays a huge role in semantic networks this small, like reported also by Beckage et al. (2011), so that more extensive longitudinal studies are usually necessary. Maybe this point can be addressed by providing some more details about Carl and Peter. Are they typical talkers? How are these two children representative of the population of normative early

talkers?

Review form: Reviewer 2 (Thomas Hills)

Is the manuscript scientifically sound in its present form?
Yes

Are the interpretations and conclusions justified by the results?
Yes

Is the language acceptable?
Yes

Is it clear how to access all supporting data?
Yes

Do you have any ethical concerns with this paper?
No

Have you any concerns about statistical analyses in this paper?
No

Recommendation?
Accept with minor revision (please list in comments)

Comments to the Author(s)
This work reports on a graph theoretic approach to syntax development in young children. The work uses the notion of chromatic number, the minimum number of colors required to properly paint a graph, to investigate the development of syntactic complexity.

I found this idea interesting and I appreciated the comparison with the null model/simulation as revealing of 'order' in syntactic development that was not previously visible.

Overall, I'm of two minds. On one side, as a new measure of graph complexity, I find chromatic number to be interesting and potentially meaningful in relation to syntax. Thus if the paper is about chromatic number and an example case, I think it succeeds (assuming it hasn't all been done before). If, on the other hand, the work is meant to teach us about syntax in a way that will be meaningful to developmental psychologists and linguists, then I think it fails. To succeed in these domains it would need to situate itself in a larger literature and explain how the new finding sits in that literature. It does not attempt to do in its present formulation beyond noting some prominent figures in the grammar/language literature. I'm somewhat indifferent and leave this to the editor to make the call. If this were reviewed only by statistical physicists, I suppose it would be fine. Indeed, if that is the framing, it is probably ready for publication pretty much as is. If it were sent out only to psychologists/linguists, I suspect few would make it past equation 2 and would find the results uninteresting. This latter group can be reached to a degree with some minor additions, unpacking the results with respect to the existing literature and offering some pointers, as I suggest below. I think that's worth doing in either case.

Specific comments:
1. More needs to be said about the null model. It seems to me there are multiple way to go about this and the method that is taken is left somewhat opaque, unless perhaps one reads citation [7],

which I didn't. For example, one could connect the words that children say by their relationships in syntactic trees. If words are nodes, which seems n obvious assumption, then figure 3 doesn't amke sense, because the number of nodes is different between the simulation and the observed data. So words aren't nodes. But since it says they are in several places throughout the paper, I'm completely lost. One could redistribute edges with or without syntactic classes, or assume some syntactic classes but not others, etc, and compare different null models. This isn't done. Perhaps that way that is done is the only meaningful way to do it, but it isn't explained why this would be true. So more needs to be said here. I'm generally in favor of competing models against one another, as it's all too easy to come up with a null model that generates data different from the observed data, but a more interesting question (to me) is what assumptions are needed to get to the observed data. That would tell us a lot.

2. I don't understand figure 4 and it seems the description definitely has at least one typo. Also, why not plot the observed and simulated together, to clearly show the differences, as in figure 3?

3. If the authors want to reach the developmentalists, they'll need to say more about what exactly chromatic number is telling us in real syntax. What's the intuition? I understand the Potts model intuition and I recognize that it measures structural information about a graph, but I don't see what it tells us about syntax. I suppose that will be for future researchers to figure out, but a comparison of more than one null model as suggested above would at least point us in a direction.

4. Little is said about previous graphical approaches to syntax, though there are quite a few running back some 30 or more years. The dominant citations are about language and syntax generally. Probabilistic (network) approaches to grammar have been found wanting in many cases (Pinker, Smolensky). I think it's important to discuss how the present approach (especially the method of network construction) is like or not like these previous approaches (see some more recent work by Kolodny et al.). I think the present work is probably most closely associated with probabilistic work like Kolodny's.

Thomas Hills

Decision letter (RSOS-181286.R0)

13-Sep-2018

Dear Dr Corominas-Murtra

On behalf of the Editors, I am pleased to inform you that your Manuscript RSOS-181286 entitled "Chromatic transitions in the emergence of syntax networks" has been accepted for publication in Royal Society Open Science subject to minor revision in accordance with the referee suggestions. Please find the referees' comments at the end of this email.

The reviewers and handling editors have recommended publication, but also suggest some minor revisions to your manuscript. Therefore, I invite you to respond to the comments and revise your manuscript.

• Ethics statement
If your study uses humans or animals please include details of the ethical approval received, including the name of the committee that granted approval. For human studies please also detail

whether informed consent was obtained. For field studies on animals please include details of all permissions, licences and/or approvals granted to carry out the fieldwork.

• Data accessibility
It is a condition of publication that all supporting data are made available either as supplementary information or preferably in a suitable permanent repository. The data accessibility section should state where the article's supporting data can be accessed. This section should also include details, where possible of where to access other relevant research materials such as statistical tools, protocols, software etc can be accessed. If the data has been deposited in an external repository this section should list the database, accession number and link to the DOI for all data from the article that has been made publicly available. Data sets that have been deposited in an external repository and have a DOI should also be appropriately cited in the manuscript and included in the reference list.

If you wish to submit your supporting data or code to Dryad (http://datadryad.org/), or modify your current submission to dryad, please use the following link:
http://datadryad.org/submit?journalID=RSOS&manu=RSOS-181286

• Competing interests
Please declare any financial or non-financial competing interests, or state that you have no competing interests.

• Authors' contributions
All submissions, other than those with a single author, must include an Authors' Contributions section which individually lists the specific contribution of each author. The list of Authors should meet all of the following criteria; 1) substantial contributions to conception and design, or acquisition of data, or analysis and interpretation of data; 2) drafting the article or revising it critically for important intellectual content; and 3) final approval of the version to be published.

All contributors who do not meet all of these criteria should be included in the acknowledgements.

We suggest the following format:
AB carried out the molecular lab work, participated in data analysis, carried out sequence alignments, participated in the design of the study and drafted the manuscript; CD carried out the statistical analyses; EF collected field data; GH conceived of the study, designed the study, coordinated the study and helped draft the manuscript. All authors gave final approval for publication.

• Acknowledgements
Please acknowledge anyone who contributed to the study but did not meet the authorship criteria.

• Funding statement
Please list the source of funding for each author.

Please note that we cannot publish your manuscript without these end statements included. We have included a screenshot example of the end statements for reference. If you feel that a given heading is not relevant to your paper, please nevertheless include the heading and explicitly state that it is not relevant to your work.

Because the schedule for publication is very tight, it is a condition of publication that you submit the revised version of your manuscript before 22-Sep-2018. Please note that the revision deadline

will expire at 00.00am on this date. If you do not think you will be able to meet this date please let me know immediately.

To revise your manuscript, log into https://mc.manuscriptcentral.com/rsos and enter your Author Centre, where you will find your manuscript title listed under "Manuscripts with Decisions". Under "Actions," click on "Create a Revision." You will be unable to make your revisions on the originally submitted version of the manuscript. Instead, revise your manuscript and upload a new version through your Author Centre.

When submitting your revised manuscript, you will be able to respond to the comments made by the referees and upload a file "Response to Referees" in "Section 6 - File Upload". You can use this to document any changes you make to the original manuscript. In order to expedite the processing of the revised manuscript, please be as specific as possible in your response to the referees. We strongly recommend uploading two versions of your revised manuscript:

1) Identifying all the changes that have been made (for instance, in coloured highlight, in bold text, or tracked changes);
2) A 'clean' version of the new manuscript that incorporates the changes made, but does not highlight them.

When uploading your revised files please make sure that you have:

1) A text file of the manuscript (tex, txt, rtf, docx or doc), references, tables (including captions) and figure captions. Do not upload a PDF as your "Main Document";
2) A separate electronic file of each figure (EPS or print-quality PDF preferred (either format should be produced directly from original creation package), or original software format);
3) Included a 100 word media summary of your paper when requested at submission. Please ensure you have entered correct contact details (email, institution and telephone) in your user account;
4) Included the raw data to support the claims made in your paper. You can either include your data as electronic supplementary material or upload to a repository and include the relevant doi within your manuscript. Make sure it is clear in your data accessibility statement how the data can be accessed;
5) All supplementary materials accompanying an accepted article will be treated as in their final form. Note that the Royal Society will neither edit nor typeset supplementary material and it will be hosted as provided. Please ensure that the supplementary material includes the paper details where possible (authors, article title, journal name).

Supplementary files will be published alongside the paper on the journal website and posted on the online figshare repository (https://rs.figshare.com/). The heading and legend provided for each supplementary file during the submission process will be used to create the figshare page, so please ensure these are accurate and informative so that your files can be found in searches. Files on figshare will be made available approximately one week before the accompanying article so that the supplementary material can be attributed a unique DOI.

Please note that Royal Society Open Science charge article processing charges for all new submissions that are accepted for publication. Charges will also apply to papers transferred to Royal Society Open Science from other Royal Society Publishing journals, as well as papers submitted as part of our collaboration with the Royal Society of Chemistry (http://rsos.royalsocietypublishing.org/chemistry).

If your manuscript is newly submitted and subsequently accepted for publication, you will be asked to pay the article processing charge, unless you request a waiver and this is approved by

Royal Society Publishing. You can find out more about the charges at http://rsos.royalsocietypublishing.org/page/charges. Should you have any queries, please contact openscience@royalsociety.org.

Once again, thank you for submitting your manuscript to Royal Society Open Science and I look forward to receiving your revision. If you have any questions at all, please do not hesitate to get in touch.

Kind regards,
Royal Society Open Science Editorial Office
Royal Society Open Science
openscience@royalsociety.org

on behalf of Dr Marcus Kaiser (Associate Editor) and Prof. Antonia Hamilton (Subject Editor)
openscience@royalsociety.org

Reviewer comments to Author:
Reviewer: 1

Comments to the Author(s)
The authors apply the chromatic number to the investigation of the growth and development of the mental lexicon of English toddlers. The chromatic number is relative to the well studied problem of graph colouring, which has been linked to other instances of problems such as SAT problems and which are massively investigated in Statistical Physics and Computer Science.

The application of the chromatic number to word learning is therefore an interesting, elegant and clever point of novelty for detecting meso-scale changes in the structure of the mental lexicon of toddlers. The authors took a great deal of effort in explaining the details behind the chromatic number also to an audience outside of the physics realm, which is the right direction for such an interdisciplinary investigation in Complexity Science.

Although the paper is nicely structured, there are some points that should be addressed by the authors before publication. In particular, my main concern is about individual variability making it quite difficult to make general statements about sudden transitions in network structure during early word learning. This crucial point should be better discussed by the authors. I believe they should underline that the syntactic spurt they detect in two children has been intensively studied by previous studies and that the current technique is capable of highlighting this pattern in those specific children. I would like for the authors to discuss some more of the representativeness of the data analysed in the current manuscript, especially considered that previous network approaches have analysed datasets of longitudinal data accounting for individual variability.

Also, in order to improve the readability of the manuscript by also language scientists from the cognitive sciences, I suggested a few references about past approaches to modelling word learning as structural transition in networks of lexical items. I would recommend for the authors to integrate these references in the manuscript in order to improve the interpretation and stress by comparison novelty of their interesting results.

The references I pointed out all agree on indicating that network modelling of early word learning is quickly becoming a “hot” topic in the community of complex systems, hence the scope and timing of the manuscript would be ideal for an interdisciplinary journal venue like Royal Society Open Science.

I hope my comments provide useful feedback from the perspective of the cognitive sciences. I would be more than happy to review again this interesting manuscript.

Massimo Stella, PhD
Fondazione Bruno Kessler, Italy
Institute for Complex Systems Simulation, University of Southampton, UK

1) Page 1, Column 1, Line 51 – The authors talk about language evolution and then structure a brief literature review of models about it. However the main scope of the paper is language acquisition. Language evolution is a different process compared to language acquisition, happening at different time scales and at different levels of a given population of language speakers. I would recommend for the authors to explicitly underline that language learning is a different process compared to language acquisition and re-focus a bit the literature review over word learning rather than on language evolution.

2) I would suggest briefly mentioning two works.
One is by cognitive scientists and uses co-occurrence networks, which partially overlap with syntactic networks, for investigating word learning in typical and late talkers:
Beckage, N., Smith, L., & Hills, T. (2011). Small worlds and semantic network growth in typical and late talkers. PloS one,6(5), e19348.
The main result of the paper is that late talkers display different co-occurrence network features compared to normative learners. Hence, syntactic networks can capture real-world patterns of language learning.

Another suggestion fitting the scope of the paper would be an approach by statistical physicists and cognitive scientists in tracking network structural changes in a multiplex network of semantic, taxonomic and phonological word-features:
Stella, M., Beckage, N. M., Brede, M., & De Domenico, M. (2018). Multiplex model of mental lexicon reveals explosive learning in humans. Scientific reports, 8(1), 2259.
The main result of the paper is that the mental lexicon of normative talkers displays an explosive phase transition around age 7 yrs, a well documented age of increased cognitive and linguistic development. This is another work showing that linguistic transitions can indeed be captured by complex networks.

Enriching the introduction with a brief mention of other relevant network approaches would increase the novelty of the approach provided by the authors of this manuscript, by contrast/comparison.

3) Page 4, Column 2, Line 37 – "First tree networks…" -> "The first tree networks…"

4) Page 4, Column 2, Last Paragraph – Please explain what "functional particles" are for the non-experts by providing some examples. Also, what do the authors mean by high "grammar flexibility"? This part of the results is particularly relevant for the paper and should be described in more detail, with some examples.

5) Page 5, Column 2, Line 49 – The measure of relevance \chi was called relative energy previously. Repeating its name here would improve the clarity of this sentence.

6) Page 5, Column 2, Last Paragraph – What do the authors mean by "clear trend towards increasing maximum clique and maximum K-core with increased relevance"? How is relevance defined? This is an important statement for justifying how the chromatic number can be a valid

proxy of “global” network features. Please reword this passage for increased clarity. The rewording has to be careful with using “global”, since even showing the presence of larger K-cores does not really imply a global structural pattern.
I imagine a simple counter-example in which a fictional network densifies its core by only adding more links, so that its maximum K-core becomes larger and K increases. However, at the same time, the network could leave its periphery completely untouched. In that case, the sign of an increasing K would not be a global estimator of structural changes in the network but rather a sign of increasing connectivity at the meso-scale level of the network core. If this was the case also in the growing syntactic network, would it be better to say that the chromatic number is an estimator of meso-scale or non-local, rather than global, network organization? The authors leave this open to speculation later on line 7 of Page 6, where they say that rather than the whole network it might be just a part of it leading to the emergence of a non-trivial K-core structure.

7) Page 6, Column 1, Line 1 – What do the authors mean by saying that the relevance of the chromatic number as a global complexity estimator is much more “feasible”? Also the term “global complexity” can be ambiguous in this context, as it might refer to specific complexity measures from the cognitive sciences rather than to addressing the structural organization of a complex network. Please reword this passage for increased clarity.

8) Page 7, Figure 4. The authors claim in the text that Figure 4 shows a well-defined non-trivial deviation between real networks and random networks. However, provided the small number of samples, it is really difficult to see this in Figure 4, between top and bottom panels. I would suggest presenting at least correlation measures or other quantitative estimates for the correlations. Also, for visual inspection it would help to produce all the panels on the same ranges and improve the size of the points. Currently, it feels difficult for the reader to reconcile the text with the figure. This point is particularly important for the very interesting results reported in the text.

9) Page 7, Column 2, Line 41 – The authors talk about combining aspects of syntax, phonology and semantics. It would be of relevance to briefly discuss again two approaches, one from the cognitive sciences and one from physicists/computer scientists.

From the cognitive sciences, it could be of relevance the approach by Dautriche and colleagues. They modelled French word acquisition in toddlers of 18 months. Dautriche and colleagues reported evidence that contrary to previous conjectures, early word learning is not dominated by simple phonological similarities but rather by a complex multidimensional combination of phonological, syntactical and semantic similarities among words. Dautriche and colleagues reported that for nouns, these multi-dimensional similarities inhibited the acquisition of new nouns while network similarities facilitated acquisition of new verbs. These facilitatory/inhibitory effects might be causing the increased structural complexity captured by the authors through the chromatic number. The reference is:
Dautriche, I., Swingley, D., & Christophe, A. (2015). Learning novel phonological neighbors: Syntactic category matters.Cognition, 143, 77-86.

From physicists, it could be of relevance the approach by Stella and colleagues, who used networks of co-occurrences, free associations, feature sharing and phonological similarities for predicting early word learning on the same dataset used by the authors. Although Stella and colleagues did not use syntactic links but rather co-occurrences as a proxy for syntactic relationships, they also showed that: (i) month 23, close to the spurt investigated by the authors, is an important “critical” phase where children start using mainly free association for word learning; (ii) syntactic relationships are important throughout early development for predicting word learning, but only when the global structure of the mental lexicon is considered, in

agreement with the message behind the chromatic number about the global structure of the mental lexicon being relevant to word acquisition. The reference is:
Stella, M., Beckage, N. M., & Brede, M. (2017). Multiplex lexical networks reveal patterns in early word acquisition in children.Scientific Reports, 7, 46730.

10) Page 8, Column 1, Line 15 – Rather than “linguistic performance” it would be more appropriate to say “linguistic proficiency”.

11) Page 8, Column 1, Line 19 – The authors indicate the chromatic number as being evidently better in underlining the presence of the syntactic spurt. However, it should be underlined that the comparison has been performed only on two children. Such a small size constitutes a problem in making general statements and should be carefully addressed by the authors. Longitudinal studies over the same CHILDES dataset used by the authors have been testing many more children. For instance, Beckage et al. (2011) investigated word learning in 66 children. Why was the current analysis limited to only 2 children? Where there data limitations? The main issue here is that individual variability plays a huge role in semantic networks this small, like reported also by Beckage et al. (2011), so that more extensive longitudinal studies are usually necessary. Maybe this point can be addressed by providing some more details about Carl and Peter. Are they typical talkers? How are these two children representative of the population of normative early talkers?

Reviewer: 2

Comments to the Author(s)
This work reports on a graph theoretic approach to syntax development in young children. The work uses the notion of chromatic number, the minimum number of colors required to properly paint a graph, to investigate the development of syntactic complexity.

I found this idea interesting and I appreciated the comparison with the null model/simulation as revealing of 'order' in syntactic development that was not previously visible.

Overall, I'm of two minds. On one side, as a new measure of graph complexity, I find chromatic number to be interesting and potentially meaningful in relation to syntax. Thus if the paper is about chromatic number and an example case, I think it succeeds (assuming it hasn't all been done before). If, on the other hand, the work is meant to teach us about syntax in a way that will be meaningful to developmental psychologists and linguists, then I think it fails. To succeed in these domains it would need to situate itself in a larger literature and explain how the new finding sits in that literature. It does not attempt to do in its present formulation beyond noting some prominent figures in the grammar/language literature. I'm somewhat indifferent and leave this to the editor to make the call. If this were reviewed only by statistical physicists, I suppose it would be fine. Indeed, if that is the framing, it is probably ready for publication pretty much as is. If it were sent out only to psychologists/linguists, I suspect few would make it past equation 2 and would find the results uninteresting. This latter group can be reached to a degree with some minor additions, unpacking the results with respect to the existing literature and offering some pointers, as I suggest below. I think that's worth doing in either case.

Specific comments:
1. More needs to be said about the null model. It seems to me there are multiple way to go about this and the method that is taken is left somewhat opaque, unless perhaps one reads citation [7], which I didn't. For example, one could connect the words that children say by their relationships in syntactic trees. If words are nodes, which seems n obvious assumption, then figure 3 doesn't amke sense, because the number of nodes is different between the simulation and the observed

data. So words aren't nodes. But since it says they are in several places throughout the paper, I'm completely lost. One could redistribute edges with or without syntactic classes, or assume some syntactic classes but not others, etc, and compare different null models. This isn't done. Perhaps that way that is done is the only meaningful way to do it, but it isn't explained why this would be true. So more needs to be said here. I'm generally in favor of competing models against one another, as it's all too easy to come up with a null model that generates data different from the observed data, but a more interesting question (to me) is what assumptions are needed to get to the observed data. That would tell us a lot.

2. I don't understand figure 4 and it seems the description definitely has at least one typo. Also, why not plot the observed and simulated together, to clearly show the differences, as in figure 3?

3. If the authors want to reach the developmentalists, they'll need to say more about what exactly chromatic number is telling us in real syntax. What's the intuition? I understand the Potts model intuition and I recognize that it measures structural information about a graph, but I don't see what it tells us about syntax. I suppose that will be for future researchers to figure out, but a comparison of more than one null model as suggested above would at least point us in a direction.

4. Little is said about previous graphical approaches to syntax, though there are quite a few running back some 30 or more years. The dominant citations are about language and syntax generally. Probabilistic (network) approaches to grammar have been found wanting in many cases (Pinker, Smolensky). I think it's important to discuss how the present approach (especially the method of network construction) is like or not like these previous approaches (see some more recent work by Kolodny et al.). I think the present work is probably most closely associated with probabilistic work like Kolodny's.

Thomas Hills

Author's Response to Decision Letter for (RSOS-181286.R0)

See Appendix A.

Decision letter (RSOS-181286.R1)

29-Oct-2018

Dear Dr Corominas-Murtra,

I am pleased to inform you that your manuscript entitled "Chromatic transitions in the emergence of syntax networks" is now accepted for publication in Royal Society Open Science.

You can expect to receive a proof of your article in the near future. Please contact the editorial office (openscience_proofs@royalsociety.org and openscience@royalsociety.org) to let us know if you are likely to be away from e-mail contact. Due to rapid publication and an extremely tight schedule, if comments are not received, your paper may experience a delay in publication.

Royal Society Open Science operates under a continuous publication model (http://bit.ly/cpFAQ). Your article will be published straight into the next open issue and this will be the final version of the paper. As such, it can be cited immediately by other researchers. As the issue version of your paper will be the only version to be published I would advise you to check your proofs thoroughly as changes cannot be made once the paper is published.

On behalf of the Editors of Royal Society Open Science, we look forward to your continued contributions to the Journal.

Kind regards,
Royal Society Open Science Editorial Office
Royal Society Open Science
openscience@royalsociety.org

on behalf of Dr Marcus Kaiser (Associate Editor) and Antonia Hamilton (Subject Editor)
openscience@royalsociety.org

Follow Royal Society Publishing on Twitter: @RSocPublishing
Follow Royal Society Publishing on Facebook:
https://www.facebook.com/RoyalSocietyPublishing.FanPage/
Read Royal Society Publishing's blog: https://blogs.royalsociety.org/publishing/

Appendix A

Dear editor of RSOS,

thank you for the careful reviewing of our manuscript and for your constructive comments. We present the new manuscript with the changes in bold face to be quickly identified. Our efforts have been focused, mainly, in trying to properly locate the paper inside the existing literature, and in providing solid justifications to our approach. Both points were specifically demanded by both reviewers. The PhD dissertation object of our preliminary email correspondence has been also added to the citation list, as requested, reference [31]:

[31] Corominas-Murtra, B. A unified approach to the emergence of complex communication. PhD dissertation (2011).

In addition, note also that we added new references, to be listed below:

[11] Karuza, E. A., Thompson-Schill, S. L. and Bassett, D. S. Trends in cognitive sciences 20 629640 (2016).

[12] Baronchelli, A., Ferrer-i Cancho, R., Pastor-Satorras, R., Chater, N. and Christiansen, M. H. Trends in cognitive sciences 17, 348360 (2013).

[16] Beckage, N., Smith, L., and Hills, T. PloS one,6(5), e19348 (2011).

18] Stella, M., Beckage, N. M., and Brede, M., Scientific Reports, 7, 46730 (2017).

[19] Stella, M., Beckage, N. M., Brede, M., and De Domenico, M. Scientific reports, 8(1), 2259 (2018).

[28] Melçuck I. Dependency Grammar: Theory and Practice.
Oxford University Press. New York (1989).

[36] Dautriche, I., Swingley, D., and Christophe, A. Cogni- tion, 143, 77-86 (2015).

[37] Kivela, M., Arenas, A., Barthelemy, M., Gleeson, J.P., Moreno Y., and Porter M. , Journal of Complex Net- works, 2, 3:203-271 (2014).

[38] Buldyrev, S.V., Parshani, R., Paul, G., Stanley H. E., and Havlin S., Nature 464, 1025-1028 (2010).

We thank the editor and both reviewers again for they really helped us to improve the quality of the manuscript. Below we detail the responses to the reviewer’s comments.

Reviewer: 1

Dear Dr. Stella,

thank you for the careful reading of our manuscript and your constructive comments. Undoubtedly, the points you raised in the revision helped us to improve the radibility of our manuscript and, more importantly, broaden the potential audience for that.

Below you have a detailed response of the points raised. In the manuscript, we have marked all the changes in bold face, to be quickly identified. We want to thank again your expert and careful analysis of our manuscript. Before starting our detailed discussion, we point out that we added several references, partially as a suggestion of your comments, to better locate our contribution both in the field of cognitive science and theory of complex networks:

[11] Karuza, E. A., Thompson-Schill, S. L. and Bassett, D. S. Trends in cognitive sciences 20 629640 (2016).

[12] Baronchelli, A., Ferrer-i Cancho, R., Pastor-Satorras, R., Chater, N. and Christiansen, M. H. Trends in cognitive sciences 17, 348360 (2013).

[16] Beckage, N., Smith, L., and Hills, T. PloS one,6(5), e19348 (2011).

18] Stella, M., Beckage, N. M., and Brede, M., Scientific Reports, 7, 46730 (2017).

[19] Stella, M., Beckage, N. M., Brede, M., and De Domenico, M. Scientific reports, 8(1), 2259 (2018).

[28] Melçuck I. Dependency Grammar: Theory and Practice.
Oxford University Press. New York (1989).

[31] Corominas-Murtra, B. A unified approach to the emergence of complex communication. PhD dissertation (2011).

[36] Dautriche, I., Swingley, D., and Christophe, A. Cogni- tion, 143, 77-86 (2015).

[37] Kivela, M., Arenas, A., Barthelemy, M., Gleeson, J.P., Moreno Y., and Porter M. , Journal of Complex Net- works, 2, 3:203-271 (2014).

[38] Buldyrev, S.V., Parshani, R., Paul, G., Stanley H. E., and Havlin S., Nature 464, 1025-1028 (2010).

Reviewer comments to Author:

1) Page 1, Column 1, Line 51 – The authors talk about language evolution and then structure a brief literature review of models about it. However the main scope of the paper is language acquisition. Language evolution is a different process compared to language acquisition, happening at different time scales and at different levels of a given population of language speakers. I would recommend for the authors to explicitly underline that language learning is a different process compared to language acquisition and re-focus a bit the literature review over word learning rather than on language evolution.

2) I would suggest briefly mentioning two works.
One is by cognitive scientists and uses co-occurrence networks, which partially overlap with syntactic networks, for investigating word learning in typical and late talkers:
Beckage, N., Smith, L., & Hills, T. (2011). Small worlds and semantic network growth in typical and late talkers. PloS one,6(5), e19348.
The main result of the paper is that late talkers display different co-occurrence network features compared to normative learners. Hence, syntactic networks can capture real-world patterns of language learning.

Another suggestion fitting the scope of the paper would be an approach by statistical physicists and cognitive scientists in tracking network structural changes in a multiplex network of semantic, taxonomic and phonological word-features:
Stella, M., Beckage, N. M., Brede, M., & De Domenico, M. (2018). Multiplex model of mental lexicon reveals explosive learning in humans. Scientific reports, 8(1), 2259.
The main result of the paper is that the mental lexicon of normative talkers displays an explosive phase transition around age 7 yrs, a well documented age of increased cognitive and linguistic development. This is another work showing that linguistic transitions can indeed be captured by complex networks.

Enriching the introduction with a brief mention of other relevant network approaches would increase the novelty of the approach provided by the authors of this manuscript, by contrast/comparison.

Thank you for the citations and the comments 1) and 2). We added the citations and a brief comment in the discussion.

3) Page 4, Column 2, Line 37 – “First tree networks…” -> “The first tree networks…”

Thanks. We corrected the typo.

4) Page 4, Column 2, Last Paragraph – Please explain what “functional particles” are for the non-experts by providing some examples. Also, what do the authors mean by high “grammar flexibility”? This part of the results is particularly relevant for the paper and should be described in more detail, with some examples.

We added a clear definition of functional particle and provided some examples.

5) Page 5, Column 2, Line 49 – The measure of relevance \chi was called relative energy previously. Repeating its name here would improve the clarity of this sentence.

There is a confusion here, since we refer to the chromatic number itself. We clarified the issue and we hope that now everything is clearer.

6) Page 5, Column 2, Last Paragraph – What do the authors mean by "clear trend towards increasing maximum clique and maximum K-core with increased relevance"? How is relevance defined? This is an important statement for justifying how the chromatic number can be a valid proxy of "global" network features. Please reword this passage for increased clarity. The rewording has to be careful with using "global", since even showing the presence of larger K-cores does not really imply a global structural pattern.
I imagine a simple counter-example in which a fictional network densifies its core by only adding more links, so that its maximum K-core becomes larger and K increases. However, at the same time, the network could leave its periphery completely untouched. In that case, the sign of an increasing K would not be a global estimator of structural changes in the network but rather a sign of increasing connectivity at the meso-scale level of the network core. If this was the case also in the growing syntactic network, would it be better to say that the chromatic number is an estimator of meso-scale or non-local, rather than global, network organization? The authors leave this open to speculation later on line 7 of Page 6, where they say that rather than the whole network it might be just a part of it leading to the emergence of a non-trivial K-core structure.

Thank you again. We reworded the whole paragraph, hoping that now everything is more understandable. We agree that it is a crucial paragraph for the paper.

7) Page 6, Column 1, Line 1 – What do the authors mean by saying that the relevance of the chromatic number as a global complexity estimator is much more "feasible"? Also the term "global complexity" can be ambiguous in this context, as it might refer to specific complexity measures from the cognitive sciences rather than to addressing the structural organization of a complex network. Please reword this passage for increased clarity.

Again, this is solved by the complete rewritting of the paragraph mentioned above.

8) Page 7, Figure 4. The authors claim in the text that Figure 4 shows a well-defined non-trivial deviation between real networks and random networks. However, provided the small number of samples, it is really difficult to see this in Figure 4, between top and bottom panels. I would suggest presenting at least correlation measures or other quantitative estimates for the correlations. Also, for visual inspection it would help to produce all the panels on the same ranges and improve the size of the points. Currently, it feels difficult for the reader to reconcile the text with the figure. This point is particularly important for the very interesting results reported in the text.

We changed the figure according to these criticisms --also raised by other reviewers. We hope that now the information is clearly conveyed to the reader.

9) Page 7, Column 2, Line 41 – The authors talk about combining aspects of syntax, phonology and semantics. It would be of relevance to briefly discuss again two approaches, one from the cognitive sciences and one from physicists/ computer scientists.

From the cognitive sciences, it could be of relevance the approach by Dautriche and colleagues. They modelled French word acquisition in toddlers of 18 months. Dautriche and colleagues reported evidence that contrary to previous conjectures, early word learning is not dominated by simple phonological similarities but rather by a complex multidimensional combination of phonological, syntactical and semantic similarities among words. Dautriche and colleagues reported that for nouns, these multi-dimensional similarities inhibited the acquisition of new nouns while network similarities facilitated acquisition of new verbs. These facilitatory/inhibitory effects might be causing the increased structural complexity captured by the authors through the chromatic number. The reference is:
Dautriche, I., Swingley, D., & Christophe, A. (2015). Learning novel phonological neighbors: Syntactic category matters.Cognition, 143, 77-86.

From physicists, it could be of relevance the approach by Stella and colleagues, who used networks of co-occurrences, free associations, feature sharing and phonological similarities for predicting early word learning on the same dataset used by the authors. Although Stella and colleagues did not use syntactic links but rather co-occurrences as a proxy for syntactic relationships, they also showed that: (i) month 23, close to the spurt investigated by the authors, is an important "critical" phase where children start using mainly free association for word learning; (ii) syntactic relationships are important throughout early development for predicting word learning, but only when the global structure of the mental lexicon is considered, in agreement with the message behind the chromatic number about the global structure of the mental lexicon being relevant to word acquisition. The reference is:
Stella, M., Beckage, N. M., & Brede, M. (2017). Multiplex lexical networks reveal patterns in early word acquisition in children.Scientific Reports, 7, 46730.

Thank you very much, for it helps to frame the discussion much better. We added these references and we briefly commented them.

10) Page 8, Column 1, Line 15 – Rather than "linguistic performance" it would be more appropriate to say "linguistic proficiency".

We performed this change.

11) Page 8, Column 1, Line 19 – The authors indicate the chromatic number as being evidently better in underlining the presence of the syntactic spurt. However, it should be underlined that the comparison has been performed only on two children. Such a small size constitutes a problem in making general statements and should be carefully addressed by the authors. Longitudinal

studies over the same CHILDES dataset used by the authors have been testing many more children. For instance, Beckage et al. (2011) investigated word learning in 66 children. Why was the current analysis limited to only 2 children? Where there data limitations? The main issue here is that individual variability plays a huge role in semantic networks this small, like reported also by Beckage et al. (2011), so that more extensive longitudinal studies are usually necessary. Maybe this point can be addressed by providing some more details about Carl and Peter. Are they typical talkers? How are these two children representative of the population of normative early talkers?

We added a justification of our choice. We also highlighted the weak points that our choice may carry on, contrasting them against the strong ones.

We thank again Dr. Massimo Stella for his constructive and enriching comments.

Dear Prof. Hills ,

thank you for the careful reading of our manuscript and your constructive comments. Undoubtedly, the points you raised in the revision helped us to improve the radibility of our manuscript and, more importantly, broaden the potential audience for that.

Below you have a detailed response of the points raised. We want to thank again your expert and careful analysis of our manuscript.

Reviewer: 2

Comments to the Author(s)
This work reports on a graph theoretic approach to syntax development in young children. The work uses the notion of chromatic number, the minimum number of colors required to properly paint a graph, to investigate the development of syntactic complexity.

I found this idea interesting and I appreciated the comparison with the null model/simulation as revealing of 'order' in syntactic development that was not previously visible.

Overall, I'm of two minds. On one side, as a new measure of graph complexity, I find chromatic number to be interesting and potentially meaningful in relation to syntax. Thus if the paper is about chromatic number and an example case, I think it succeeds (assuming it hasn't all been done before). If, on the other hand, the work is meant to teach us about syntax in a way that will be meaningful to developmental psychologists and linguists, then I think it fails. To succeed in these domains it would need to situate itself in a larger literature and explain how the new finding sits in that literature. It does not attempt to do in its present formulation beyond noting some prominent figures in the grammar/ language literature. I'm somewhat indifferent and leave this to the editor to make the call. If this were reviewed only by statistical physicists, I suppose it would be fine. Indeed, if that is the framing, it is probably ready for publication pretty much as is. If it were sent out only to psychologists/linguists, I suspect few would make it past equation 2 and would find the results uninteresting. This latter group can be reached to a degree with some minor additions, unpacking the results with respect to the existing literature and offering some pointers, as I suggest below. I think that's worth doing in either case.

Specific comments:

1. More needs to be said about the null model. It seems to me there are multiple way to go about this and the method that is taken is left somewhat opaque, unless perhaps one reads citation [7], which I didn't. For example, one could connect the words that children say by their relationships in syntactic trees. If words are nodes, which seems an obvious assumption, then figure 3 doesn't make sense, because the number of nodes is different between the simulation

and the observed data. So words aren't nodes. But since it says they are in several places throughout the paper, I'm completely lost. One could redistribute edges with or without syntactic classes, or assume some syntactic classes but not others, etc, and compare different null models. This isn't done. Perhaps that way that is done is the only meaningful way to do it, but it isn't explained why this would be true. So more needs to be said here. I'm generally in favor of competing models against one another, as it's all too easy to come up with a null model that generates data different from the observed data, but a more interesting question (to me) is what assumptions are needed to get to the observed data. That would tell us a lot.

Thanks for raising this point. We added a new paragraph justifying the choice of the null model. It would seem natural to go for a standard graph randomization techinque, however, the graph here is a surrogate of a deeper phenomena. Our choice has been, therefore, to create a corpus of a speaker who, sharing the statistics of sentence length and number of utterances, lacks any syntactic structure and, then, build the network upon this 'random corpus'. Under our viewpoint, this null model, even more elaborated than others, grasps the essence of the problem we are dealing with. We hope that the wider justification we provide in the revised version can clarify the doubts raised before.

2. I don't understand figure 4 and it seems the description definitely has at least one typo. Also, why not plot the observed and simulated together, to clearly show the differences, as in figure 3?

We changed the figure such that now everything is easier to understand.

3. If the authors want to reach the developmentalists, they'll need to say more about what exactly chromatic number is telling us in real syntax. What's the intuition? I understand the Potts model intuition and I recognize that it measures structural information about a graph, but I don't see what it tells us about syntax. I suppose that will be for future researchers to figure out, but a comparison of more than one null model as suggested above would at least point us in a direction.

Thanks for raising this point, which helps us to reach a wider audience. We added a paragraph in the introduction motivating the use of the chromatic number as a suitable measure for syntax development.

4. Little is said about previous graphical approaches to syntax, though there are quite a few running back some 30 or more years. The dominant citations are about language and syntax generally. Probabilistic (network) approaches to grammar have been found wanting in many cases (Pinker, Smolensky). I think it's important to discuss how the present approach (especially the method of network construction) is like or not like these previous approaches (see some more recent work by Kolodny et al.). I think the present work is probably most closely associated with probabilistic work like Kolodny's.

We better motivated our network construction, specifically we explain that the underlying method is based in the so called dependency grammar, developed in reference [28]. In addition, we added more references in order to better locate

our work both in the conginive sciences and network approaches to complex systems. Specifically, we added the following citations:

[11] Karuza, E. A., Thompson-Schill, S. L. and Bassett, D. S. Trends in cognitive sciences 20 629640 (2016).

[12] Baronchelli, A., Ferrer-i Cancho, R., Pastor-Satorras, R., Chater, N. and Christiansen, M. H. Trends in cognitive sciences 17, 348360 (2013).

[16] Beckage, N., Smith, L., and Hills, T. PloS one,6(5), e19348 (2011).

18] Stella, M., Beckage, N. M., and Brede, M., Scientific Reports, 7, 46730 (2017).

[19] Stella, M., Beckage, N. M., Brede, M., and De Domenico, M. Scientific reports, 8(1), 2259 (2018).

[28] Melçuck I. Dependency Grammar: Theory and Practice.
Oxford University Press. New York (1989).

[31] Corominas-Murtra, B. A unified approach to the emergence of complex communication. PhD dissertation (2011).

[36] Dautriche, I., Swingley, D., and Christophe, A. Cogni- tion, 143, 77-86 (2015).

[37] Kivela, M., Arenas, A., Barthelemy, M., Gleeson, J.P., Moreno Y., and Porter M. , Journal of Complex Net- works, 2, 3:203-271 (2014).

[38] Buldyrev, S.V., Parshani, R., Paul, G., Stanley H. E., and Havlin S., Nature 464, 1025-1028 (2010).

We thank again Prof. Thomas Hills for his constructive and enriching comments.
